# Editorial: Solanaceae VII: Biology, Genetics, and Evolution

**DOI:** 10.3389/fgene.2022.932421

**Published:** 2022-06-23

**Authors:** Péter Poczai, Nunzio D’Agostino, Rocio Deanna, Ezio Portis

**Affiliations:** ^1^ Finnish Museum of Natural History, University of Helsinki, Helsinki, Finland; ^2^ Museomics Research Group, Department of Biosciences, Viikki Plant Science Centre (ViPS), University of Helsinki, Helsinki, Finland; ^3^ Institute of Advanced Studies Kőszeg (iASK), Kőszeg, Hungary; ^4^ Department of Agricultural Sciences, University of Naples Federico II, Portici, Italy; ^5^ Department of Ecology and Evolutionary Biology, University of Colorado, Boulder, CO, United States; ^6^ Instituto Multidisciplinario de Biología Vegetal (IMBIV, CONICET-UNC), Córdoba, Argentina; ^7^ Department of Agricultural, Forest and Food Sciences (DISAFA), Plant Genetics, University of Turin, Grugliasco, Italy

**Keywords:** anthocyanin biosynthesis, biosphere reserves, biotic/abiotic stresses, conservation biogeography, endogenous hormones, fruit set, genome-wide association studies, karyotype

The Solanaceae family, also referred to as nightshades or the tomato family, includes a wide variety of morphologies and habits. It has a distinct evolutionary history driven by natural selection, among other processes, which has resulted in unique adaptations and rewarding biochemical pathways for pollinators. The Solanaceae family contains 99 genera and approximately 2,700 species, including economically important crops such as potatoes, tomatoes, peppers, and eggplants (Figure 1). It also includes several other underutilized nutrient-rich minor crops, such as turkey berry, African nightshades, or scarlet eggplant, which play a key role in the sustainability of local communities both in the Western and Eastern Hemisphere. Many other solanaceous plants are vital sources of medicinal drugs, folk remedies, and hallucinogens, while wild and cultivated species, such as tobacco, are commonly used as stimulants. Several groups, such as petunia seasides, angel’s trumpets, butterfly flowers, and Jerusalem cherry have been grown as ornamentals for centuries due to the aesthetic appeal of their flowers and berries.

**FIGURE 1:**
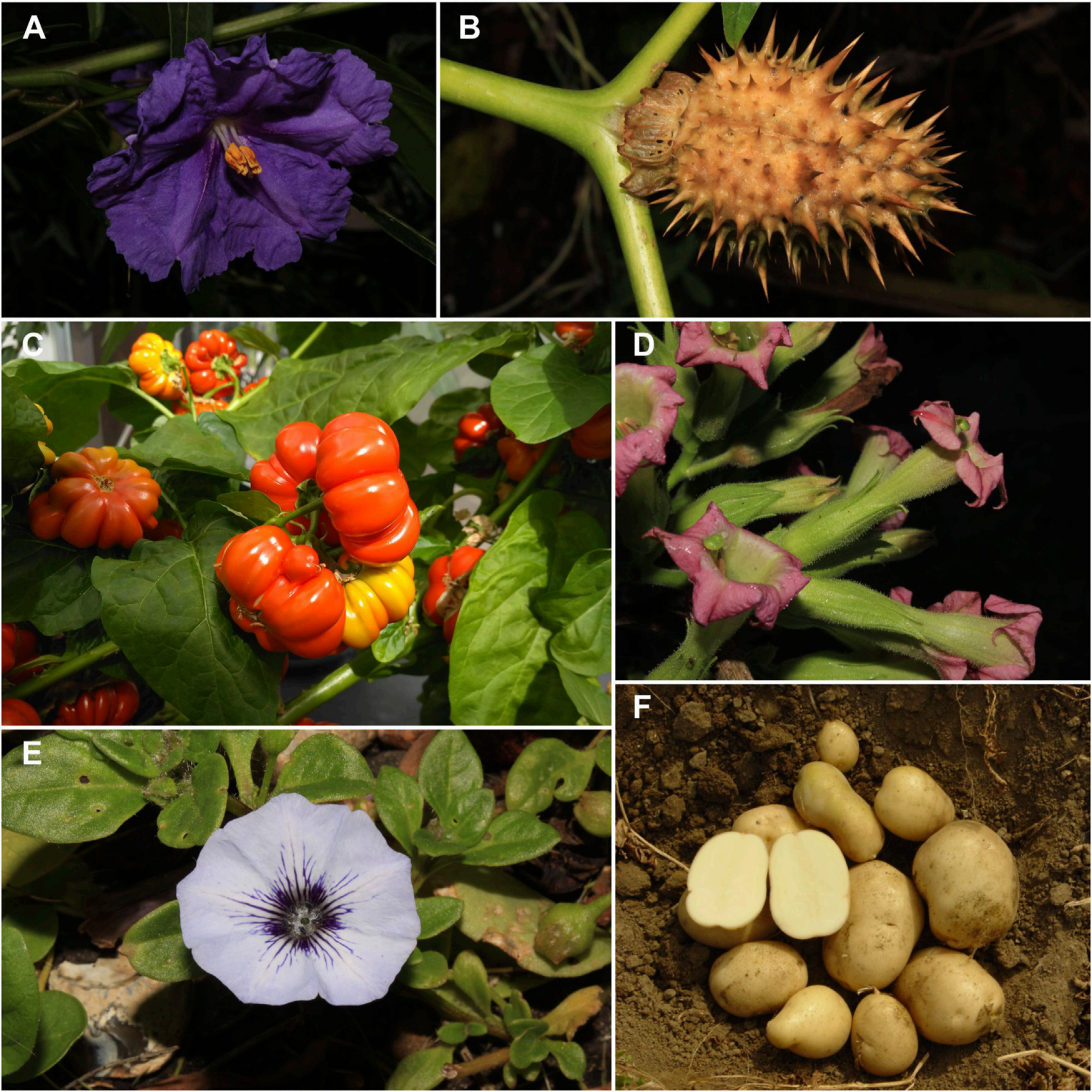
Diversity of Solanaceae crops, poisonous weeds, and relatives. **(A)** *Solanum laciniatum* Aiton, **(B)** *Datura stramonium* L., **(C)** *Solanum lycopersicum* L., **(D)** *Nicotiana tabacum* L., **(E)** *Nolana humifusa* (Gouan) I.M.Johnst., **(F)** *Solanum tuberosum* L. A-B, D-E photographs by R. Deanna at Chelsea Physics Garden; C by P. Poczai; F by D. Carputo.

The declining resilience of ecosystems experiencing biodiversity loss has increased the urgency of promoting the sustainable conservation and use of globally important solanaceous plants. The toolbox available to scientists and breeders is increasingly diversifying to mitigate the emerging challenges imposed by climate change while honoring biodiversity. This diversification could lead to major transformations towards sustainability and how our global society functions and interacts with natural ecosystems. Recent developments in high-throughput sequencing technologies, data science, and internationally open biodiversity data infrastructures are effectively “linking biodiversity with omics” for a better understanding of this important family, as envisioned nearly two decades ago.

The list of Solanaceae with full or publicly available high quality and draft genomes is becoming richer, which allows the “sequence space” of most Solanaceae to be explored to: improve access to gene inventory catalogs; broaden our understanding of phylogeny, biogeography, and taxonomy; identify beneficial alleles for breeding; reveal the breeding potential of wild germplasm; study the mechanisms of gene expression; explore the architecture and organization of chromosomes, capturing the extent of genomic variation; use sequence-based information to perform large-scale genetic (bio)diversity studies; identify the genomic loci underlying key genes and selection sweeps; disentangle metabolic pathways; learn about plant-microbe interaction, (a)biotic stress responses, and defense mechanisms against pathogens and pests.

## Advances in Biology and Evolution

For a solanaceous plant to survive, its growth, pollination, and development must be carefully coordinated by a variety of internal and external signals. Controlling gene expression is crucial for the regulation of biological processes including body planning, development, differentiation, and response to numerous environmental changes. Genome expression and modulation begin with transcription, which is the first stage in this process. To better understand how Solanaceae regulate gene expression, transcriptional regulators of the myeloblastosis (*MYB*) transcription factor family were studied in *Capsicum chinense* Jacq. by Islam et al. Their study revealed that several *MYB* genes are involved in determining fruit shape and size, creating opportunities for further trait improvement. Borràs et al. highlighted the different roles of two NAC homologs in *Capsicum* and discussed the complex role of NACs as transcriptional switches in the response to drought stress. The research reported by Wang et al. provided a genome-wide characterization and anthocyanin-related expression analysis of the B-BOX gene family in *C. annuum* L., providing a foundation for further investigation of the CaBBX genes involved in the anthocyanin synthesis mechanisms and development in pepper. The ubiquitous transcription factor nuclear factor Y (NF-Y) has been identified by Liu et al. as a key regulator in fruit development, abiotic stress tolerance, and anthocyanin biosynthesis in potato. In addition, Li et al. provided a characterization of members of the SRO gene family in tomato, how widely expressed they are in different tissues, and their response to high temperature and salt stress, while mediating the hormone regulatory network.

Pollination is typically an uncertain process that depends on many factors that determine the reproductive success of Solanaceae. Self-incompatibility (SI), described by Charles Darwin (1809–1882) in “*The effects of cross and self-fertilization in the vegetable kingdom (1876)*,” *i*s one of these interesting factors often observed in solanaceous plants. Although it was demonstrated early on that self-fertility follows Mendelian inheritance, the underlying molecular basis of SI remained a black box until the mid-1980s. This mechanism was explored in wild tomatoes by Broz et al. who have highlighted that S-RNase expression is more dynamic than previously thought and that changes in expression can impact various reproductive barriers within or between natural populations.

When a solanaceous plant senses an external stimulus, hormones play a key role in its reaction. From blooming through fruit setting and ripening to phototropism and leaf fall, plant hormones affect all aspects of Solanaceae life. Every cell of a tomato plant, for example, may be able to produce these hormones, but this is not a certainty. Therefore, information about the molecular mechanisms underlying hormone synthesis, transport, and signaling is critical to understanding and later accurately engineering these pathways for crop improvement. Hormones have a critical effect on pollination and fruit development, which is pivotal for horticultural production. Endogenous hormones in the ovaries of parthenocarpic and non-parthenocarpic tomato lines were examined to investigate fruit development without pollination. The investigation by Zhang et al. highlighted that auxin plays a prominent role in controlling fruit set in a self-pollinating tomato, while other hormones are integrated in a synergistic or antagonistic way within this process.

Moreira-Munoz et al. described the diversity of Solanaceae in southern South America, assessing conservation gaps in relation to protected areas. They identified micro-hotspots of species richness and reiterated the need for conservation plans in Argentina and Chile. Ramírez-Ojeda et al. determined the most important edaphoclimatic descriptors of wild tomato species and their closely related species along with their natural geographic range in South America. Their work can be used to study the impact of climate change and anthropic activities across the range of these species. Deanna et al. summarized the current understanding of cytogenetics in Solanaceae, its applications, and prospects for making progress in fundamental systematic botany and plant evolution. The information compiled highlights the importance of basic chromosome features in understanding the evolution of the family, especially in delimiting clades.

## Genetics of Disease Resistance and Resources to Accelerate Breeding

Solanaceous plants have evolved sophisticated defense mechanisms to combat a variety of pests and diseases. Plant immune systems rely on their capacity to identify and transduce signaling molecules, as well as to respond defensively via pathways involving several genes and their products. Lai et al. performed GWAS on a collection of nearly 100 tobacco accessions to identify QTNs (quantitative trait nucleotides) associated with tobacco bacterial wilt resistance. Association tests returned 38 stable novel QTNs, which allowed the identification of superior alleles for the development of tobacco varieties highly resistant to *Ralstonia solanacearum*. Dubey et al. proceeded with the *in silico* identification and characterization of all members of the gene subfamily TNL (TIR-NBS-LRR) in the potato genome. Following quantitative RT-PCR, one of the genes, namely StTNLC7G2, showed induced expression after *Alternaria solani* attack and was thus functionally characterized. The results showed that StTNLC7G2 is responsible for the induction of reactive oxygen species and plays a key role in the hypersensitive response during plant defense.

Wild relatives of cultivated solanaceous crops are often sources of natural plant disease resistance. These plant genetic resources offer a wide variety of untapped traits that are beneficial for breeding programs. Mangino et al. described an 8-way MAGIC population developed using seven cultivated *Solanum melongena* L. accessions and a wild relative (*S. incanum* L.) as founders. The population, which includes 420 S3 individuals, has been genotyped and phenotyped for anthocyanin-related traits. Association tests returned strong signals for two MYB genes and a COP1 gene, all directly involved in the anthocyanin biosynthesis pathway. Gong et al. reviewed the germplasm resources, their collection strategies for breeding, and molecular-assisted breeding progress in *Lycium* (goji), a widely spread species of the arid to semiarid environments of Eurasia, Africa, North and South America, among which most species have high potential as healthy food. They also discussed recent strategies for QTL mapping and protocols for trait detection, by providing a number of traits (275 agronomic and 870 metabolic) in various organs based on reference studies on *Lycium*, tomato, and other Solanaceae species.

In summary, the current Research Topic (RT) provides further insight into understanding the important mechanisms of the biology of solanaceous plants in terms of biodiversity, plant growth, pollination, cytogenetics, fruit development, and resistance to pathogens.

